# Climate change hotspots in the CMIP5 global climate model ensemble

**DOI:** 10.1007/s10584-012-0570-x

**Published:** 2012-08-25

**Authors:** Noah S. Diffenbaugh, Filippo Giorgi

**Affiliations:** 1 Department of Environmental Earth System Science and Woods Institute for the Environment, Stanford University, 473 Via Ortega, Stanford, CA 94305-4216 USA; 2 Earth System Physics Section, Abdus Salam International Centre for Theoretical Physics, Trieste, Italy

## Abstract

We use a statistical metric of multi-dimensional climate change to quantify the emergence of global climate change hotspots in the CMIP5 climate model ensemble. Our hotspot metric extends previous work through the inclusion of extreme seasonal temperature and precipitation, which exert critical influence on climate change impacts. The results identify areas of the Amazon, the Sahel and tropical West Africa, Indonesia, and the Tibetan Plateau as persistent regional climate change hotspots throughout the 21st century of the RCP8.5 and RCP4.5 forcing pathways. In addition, areas of southern Africa, the Mediterranean, the Arctic, and Central America/western North America also emerge as prominent regional climate change hotspots in response to intermediate and high levels of forcing. Comparisons of different periods of the two forcing pathways suggest that the pattern of aggregate change is fairly robust to the level of global warming below approximately 2 °C of global warming (relative to the late-20th-century baseline), but not at the higher levels of global warming that occur in the late-21st-century period of the RCP8.5 pathway, with areas of southern Africa, the Mediterranean, and the Arctic exhibiting particular intensification of relative aggregate climate change in response to high levels of forcing. Although specific impacts will clearly be shaped by the interaction of climate change with human and biological vulnerabilities, our identification of climate change hotspots can help to inform mitigation and adaptation decisions by quantifying the rate, magnitude and causes of the aggregate climate response in different parts of the world.

**Electronic supplementary material:**

The online version of this article (doi:10.1007/s10584-012-0570-x) contains supplementary material, which is available to authorized users.

## Introduction

It is now established not only that human activities have been the primary cause of observed global warming (IPCC 2007), but also that the climate system is already committed to further global warming arising from physical (e.g., (Meehl et al. 2005)), biogeochemical (e.g., (Jones et al. 2010)), socio-political (e.g., (Matthews and Weaver 2010)) and infrastructural (e.g., (Davis et al. 2010)) inertia. In addition, continued unconstrained increases in greenhouse gas emissions are likely to cause global warming that substantially exceeds the internationally agreed-upon target (UNFCCC 2009) of 2 °C above the pre-industrial baseline (e.g., (Matthews et al. 2009; Meinshausen et al. 2009)). Decisions about how best to adapt to committed warming and about what level of warming to target in order to avoid unacceptable climate change require understanding of the pattern and magnitude of the regional and local response to different levels of radiative forcing. Although in many areas of the world the impacts of climate change are likely to be determined by highly-localized physical, biological and human factors, quantifying the magnitude of integrated change across a suite of physical climate indicators can help to identify climate change “hotspots” that show the strongest and most robust aggregated response to global-scale warming.

Giorgi (2006) quantified sub-continental-scale climate change hotspots in the late-21st-century period of Phase 3 of the Coupled Model Intercomparison Project (CMIP3). A weighting of changes in mean and variability of seasonal temperature and precipitation revealed the Mediterranean, the northern hemisphere high-latitude regions, and Central America as the most prominent hotspots (Giorgi 2006). Other aggregations of multi-dimensional climate change include integration of sea-level-rise vulnerability into the Giorgi index (Diffenbaugh et al. 2007a), summation of the number of seasons exceeding different temperature and precipitation thresholds (Baettig et al. 2007), use of statistical metrics of the distance traveled in multi-dimensional climate space (Williams et al. 2007; Diffenbaugh et al. 2008), and use of statistical metrics of the magnitude and/or rate of climate change experienced by particular biological categories (Loarie et al. 2009; Ackerly et al. 2010; Beaumont et al. 2011; Sandel et al. 2011). Given the availability of a new generation of global climate model simulations that comprise the CMIP5 ensemble (Taylor et al. 2012), we quantify the transient emergence of global hotspot patterns using a statistical metric of aggregate multi-dimensional climate change. This metric extends the statistical approach of Diffenbaugh et al. (2008) to also include measures of extreme seasonal temperature and precipitation, which are particularly important for climate change impacts.

## Methods

### Models

We quantify climate change hotspots in the 2016–2035, 2046–2065, and 2080–2099 periods of the CMIP5 RCP8.5 and RCP4.5 simulations. RCP8.5 and RCP4.5 diverge dramatically over the 21st century, reaching greenhouse gas concentrations of >1370 and ~650 ppm CO_2_-e (Moss et al. 2010), respectively, by the year 2100, along with radiative forcing of ~8.5 and ~4.5 W/m^2^ (Moss et al. 2010), and median global warming of 4.9 and 2.4 °C above the pre-industrial baseline (Rogelj et al. 2012). The global warming in RCP8.5 and RCP4.5 most closely match that in the A1FI and B1 SRES scenarios, respectively (Rogelj et al. 2012).

The suite of available simulations includes realizations from 20 models, including 86 realizations of the baseline period (1986–2005), and 51 realizations of the 21st century in both the RCP8.5 and RCP4.5 pathways (Table S1). Following Giorgi (2006), Diffenbaugh et al. (2007a), and Diffenbaugh et al. (2008), our analysis is carried out after first interpolating the output from each model to a common 1-degree geographical grid.

Further details of the CMIP5 simulations are provided in the Supplemental Information (SI).

### Hotspot quantification

Following Diffenbaugh et al. (2008), we use the Standard Euclidean Distance (SED) to quantify the total change in multi-dimensional climate space between the present and future periods:

Eq.1 $$ {\text{SE}}{{\text{D}}_{{total}}} = {\left( {{\sum_v}{\text{SE}}{{\text{D}}_v}} \right)^{{{1}/{2}}}} $$

for

Eq.2 $$ {\text{SE}}{{\text{D}}_v} = { }{\left( {{ }{{{{\text{abs}}\left( {{\Delta_v}} \right)}} \left/ {{{ \max }{{\left[ {{\text{abs}}\left( {{\Delta_v}} \right)} \right]}_{{ij}}}}} \right.}} \right)^{{2}}} $$

where abs(*∆*_*v*_) is the absolute value of change in climate indicator *v* at each grid point between the present and future periods, and max[abs(∆_*v*_)]_*ij*_ is the maximum land-grid-point absolute value change in climate indicator *v* over all land grid points *ij* in the 2080–2099 period of RCP8.5 (i.e., the change in each period is normalized to the maximum change in the 2080–2099 period of RCP8.5). By scaling to the maximum change in the highest forcing period, our approach yields a relative metric of aggregate climate change that can be directly compared between geographic areas, forcing pathways, and time periods within a forcing pathway.

We include 7 climate indicators from each of four seasons (DJF, MAM, JJA, SON), yielding 28 total dimensions at each grid point. The climate indicators are: absolute change in mean surface air temperature, fractional change in mean precipitation, fractional change in interannual standard deviation of de-trended surface air temperature, fractional change in interannual coefficient of variation of de-trended precipitation, frequency of occurrence of seasons above the baseline maximum seasonal surface air temperature, frequency of occurrence of seasons below the baseline minimum seasonal precipitation, and frequency of occurrence of seasons above the baseline maximum seasonal precipitation. We calculate the simulated change in each variable using the ensemble mean of that variable in the baseline and future periods.

Our aggregate metric only considers land grid points north of 60°S. In order to treat the change in each of the 28 dimensions equally in the SED calculation, we normalize the change in each climate indicator by first calculating the absolute value of change at each land grid point and then dividing by the largest grid-point absolute-value change that occurs at any land grid point north of 60°S in the 2080–2099 period of RCP8.5. We then calculate the SED at each land grid point by first squaring each of the normalized values, then summing the squared values, and then taking the square root of the sum.

Further details of the hotspot quantification are provided in the SI.

## Results and discussion

**Fig. 1:**
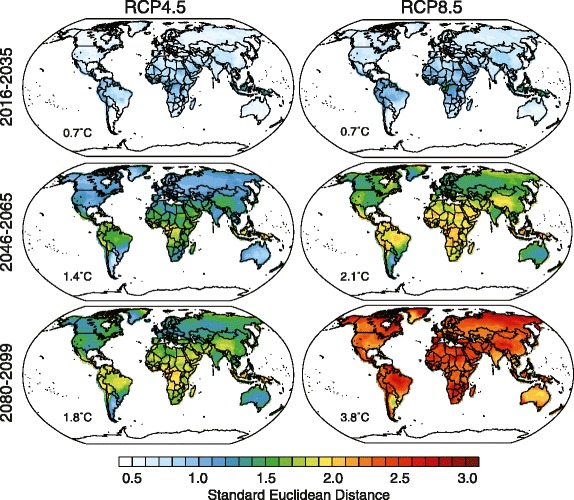
The relative aggregate climate change between the 1986–2005 period and the 2016–2035, 2046–2065 and 2080–2099 periods of RCP8.5 and RCP4.5. The aggregate climate change is calculated using the Standard Euclidean Distance (SED) across the 28-dimensional climate space formed by 7 climate indicators in each of 4 seasons. Prior to calculating the SED, the absolute values of change in each climate indicator are normalized to the maximum global absolute value in the 2080–2099 period of RCP8.5. Only land grid points north of 60°S are used in the normalization. The median global temperature change above the late 20th century baseline is given from Rogelj et al. (2012) in the lower left corner of each panel

The hotspot patterns for the three future time periods of RCP8.5 and RCP4.5 are shown in Fig. 1. The dominant global hotspot pattern emerges early in the 21st century, with areas of the Amazon, the Sahel and tropical West Africa, Indonesia, and the Tibetan Plateau emerging early in the 21st century and exhibiting relatively high aggregate climate change in all three periods of both forcing pathways. In addition, areas of southern Africa, the Mediterranean, the Arctic, and Central America/western North America emerge at intermediate and/or high levels of forcing, while areas of southern South America, Australia, the Indian Peninsula, and East Asia exhibit relatively small – but increasing – aggregate climate change throughout the 21st century (Fig. 1).

**Fig. 2:**
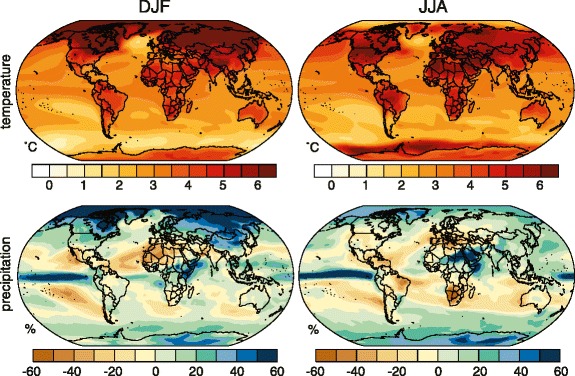
The change in December-January-February (DJF) and June-July-August (JJA) surface air temperature and precipitation between the 1986–2005 period and the 2080–2099 period of RCP8.5 in the CMIP5 ensemble

**Fig. 3:**
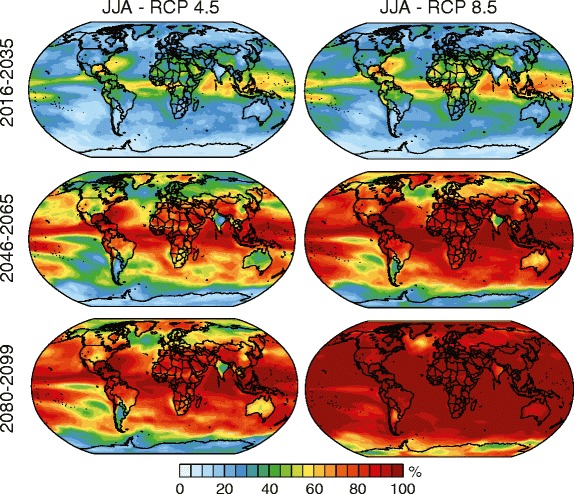
The occurrence of the 1986–2005 maximum June-July-August (JJA) seasonal temperature in the 2016–2035, 2046–2065 and 2080–2099 periods of RCP8.5 (*left*) and RCP4.5 (*right*). The panels show the absolute occurrences as the percent of years in each 20-year period. The frequency of occurrence of the 1986–2005 maximum JJA seasonal temperature value is, by definition, 5 % at each grid point during the 20-year 1986–2005 period

The aggregate hotspot patterns reflect the pattern and magnitude of changes in the mean, variability and extremes of seasonal temperature and precipitation (Figs. 2, 3, S1 and S2). The regions that show the strongest aggregate climate changes exhibit large values of relative change in a number of different climate indicators (Fig. 2 and S2). For example, in the 2080–2099 period, the Amazon exhibits areas of relatively large changes in JJA mean precipitation (Figs. 2 and S2), DJF and SON precipitation variability (Fig. S2), DJF and MAM temperature variability (Fig. S2), and DJF, MAM, JJA and SON extreme dry seasons (Fig. S2). Likewise, northeast Eurasia exhibits areas of relatively large changes in DJF, MAM and SON mean temperature (Figs. 2 and S2), DJF and MAM mean precipitation (Figs. 2 and S2), and DJF, MAM, JJA and SON extreme wet seasons (Fig. S2).

Comparisons of different periods of the two forcing pathways suggest that the pattern of aggregate change is fairly robust to the level of global warming below approximately 2 °C of global warming (relative to the late-20th-century baseline), but not at the higher levels of global warming that occur in the late-21st-century period of the RCP8.5 pathway (Fig. 1). For example, the tropical regions exhibit the greatest relative change throughout the RCP4.5 pathway, with much of central Africa exhibiting increases in aggregate change from approximately 1.2 during the 2016–2035 period of RCP4.5, to approximately 1.9 during the 2046–2065 period of RCP4.5, to approximately 2.1 during the 2080–2099 period of RCP4.5. In contrast, the high latitudes consistently exhibit smaller relative aggregate change than the tropics throughout the RCP4.5 pathway, with broad areas of the Arctic exhibiting increases in aggregate change from approximately 0.7 during the 2016–2035 period of RCP4.5, to approximately 1.3 during the 2046–2065 period of RCP4.5, to approximately 1.6 during the 2080–2099 period of RCP4.5. The pattern of greatest relative aggregate change occurring over tropical regions is also seen during the 2046–2065 period of RCP8.5, when median global warming is larger than in the 2080–2099 period of RCP4.5 (Rogelj et al. 2012)). However, the highest values of relative aggregate change occur much more broadly during the late-21st-century period of RCP8.5, with central Africa and Indonesia both exhibiting lower aggregate values (up to 2.5) than the Arctic (up to 3.0), the Mediterranean (up to 2.9), the Sahel (up to 2.9), the Amazon (up to 2.8), Southern Africa (up to 2.8), and Tibet (up to 2.8).

The apparent acceleration of relative aggregate climate change over areas of the extra-tropics at high levels of global warming (Fig. 1) results in part from the fact that intensification of extreme hot season occurrence emerges most strongly over the tropics in the early- and mid-21st century periods of both forcing pathways, but emerges equally strongly over most areas of the globe by the late 21st century of RCP8.5 (Figs. 3 and S3). For example, the occurrence of extreme hot seasons over tropical Africa, tropical South America, and Indonesia is at least twice as large as the occurrence over most mid- and high-latitude areas in the 2016–2035 period. This regional contrast is almost entirely eliminated in the 2080–2099 period of RCP8.5 as extreme hot seasons become common over all inhabited land areas, meaning that differences in the relative metric are instead shaped by other climate indicators.

In addition to climate change hotspots, our metric also identifies areas that exhibit relatively small aggregate response to global warming. For example, southern South America and the Indian Peninsula consistently exhibit reduced magnitude of change in mean, variability and extremes of temperature and precipitation relative to other areas of the globe (Figs. 3 and S2), suggesting that those regions could face reduced risk of increasing climate-related stresses. However, areas that exhibit relatively low aggregate change could still be vulnerable to climate changes that occur in response to continued global warming. For example, in the 2080–2099 period of RCP8.5, at least 65 % of all seasons are extremely hot over all land areas, and at least 80 % of all seasons are extremely hot over most land areas (Figs. 3 and S2). Frequent extreme heat could have substantial impacts on natural and human systems (e.g., (Ciais et al. 2005; White et al. 2006; Schlenker and Roberts 2009; Toomey et al. 2011; Diffenbaugh et al. 2012)), regardless of the global pattern of relative aggregate climate change.

Although the hotspot patterns appear to be robust to varying levels of greenhouse forcing (Fig. 1), the results are subject to other sources of uncertainty. For example, although the CMIP5 ensemble is unprecedented in its scope, the number of models and of realizations is insufficient to span the full range of uncertainty in global climate sensitivity and regional response to global warming (Taylor et al. 2012). As a result, although we have attempted to give all models equal contribution to the ensemble mean (see SI), the results reported here could be sensitive to the number of models included in the ensemble, and to the number of realizations of each model. In addition, internal climate system variability could overwhelm the identified climate change patterns for time scales that are shorter than the multi-decadal scales explored here, meaning that individual decadal or sub-decadal periods could show different patterns of aggregate climate anomalies.

## Conclusions

Our statistical metric of multi-dimensional climate change identifies areas of the Amazon, the Sahel and tropical West Africa, Indonesia, and the Tibetan Plateau as regional climate change hotspots that persist throughout the 21st century of both the RCP8.5 and RCP4.5 forcing pathways. Comparisons of different periods of the two forcing pathways suggest that the magnitude of aggregate climate change varies relatively linearly with the magnitude of global warming below approximately 2 °C of global warming (relative to the late-20th-century baseline). However, our metric also identifies areas of southern Africa, the Mediterranean, the Arctic, and Central America/western North America as prominent regional climate change hotspots that emerge in response to higher levels of forcing, with areas of southern Africa, the Mediterranean, and the Arctic exhibiting late-21st-century climate change that is as large as that exhibited by the persistent hotspots. It is possible that non-linear processes such as snow-albedo feedbacks and soil-precipitation interactions (e.g., (Hall 2004; Diffenbaugh et al. 2005; Seneviratne et al. 2006; Diffenbaugh et al. 2007b; Quesada et al. 2012)) contribute to the emergence of these accelerating hotspots.

The primary discrepancies between our hotspot identification and that of Giorgi (2006) occur in the tropics, with the tropical hotspots in our analysis being strongly influenced by increases in extreme seasons, which were not included in the analysis of Giorgi (2006). The pattern of high and low aggregated change is quite similar to that reported by Baettig et al. (2007), who analyzed the occurrence of annual- and seasonal-scale extreme temperature and precipitation in the CMIP3 ensemble, and with the pattern of “novel” and “disappearing” climates reported by Williams et al. (2007), who used the SED to analyze changes in seasonal temperature and precipitation as a fraction of the baseline variability. Our reported pattern is also reflective of that reported by Beaumont et al. (2011), who emphasized an extreme temperature metric similar to ours, resulting in prominent tropical hotspots. In contrast, our aggregated climate change patterns show less agreement with the climate change “velocity” patterns of Sandel et al. (2011) and Loarie et al. (2009), which were focused on the absolute rate of change of mean annual temperature, and showed greater responsiveness at mid and high latitudes.

Our hotspot analysis has a number of possible applications. For instance, the identified patterns of aggregate change provide a clear “fingerprint” for climate change detection and attribution studies. In addition, the pattern and relative magnitude of aggregate change at different levels of global warming can inform mitigation decisions by helping to evaluate the relative magnitude of aggregate climate change that different regions face within a given global warming target. Likewise, the hotspot analysis can also inform mitigation decisions by identifying particular regions that exhibit increased relative change at higher levels of global warming, as in the case of southern Africa, the Arctic and the Mediterranean in our analysis. Further, the patterns of aggregate change indicate which regions are likely to face the greatest near-term climate change that is already committed through climate system inertia. For instance, our analysis suggests that the most immediate emerging climate stresses are likely to be associated with extreme heat, with the most immediate intensification likely to occur in tropical Africa, Indonesia, and parts of the Amazon.

It is important to stress that our analysis is constructed to quantify the relative responsiveness of physical climate in different regions, and not to imply the relative magnitude of climate change impacts or of vulnerability to climate change. However, because our metric quantifies the distance traveled in multi-dimensional climate space, it can provide a compact indicator of potential risk of climate change impacts. For example, in many systems, the impacts of climate change are likely to be greatest where there are multiple environmental stresses (e.g., (Parsons 1990; Helmuth et al. 2005; Smith et al. 2009)). The impacts of multiple stresses often result from interactions that would not be predicted from the individual stresses themselves (e.g., (Vinebrooke et al. 2004; Niinemets 2010)). As one example, analysis of the severe droughts that struck the Amazon in 2005 and 2010 shows that severe heat exacerbated the effects of dry soils to create losses in above-ground biomass that were not explained by moisture stress alone (Toomey et al. 2011). Our analysis suggests that for global warming of ~3.8 °C, the Amazon faces combined risk of extreme heat and extreme drought that is greater than any other region. Therefore, in addition to identifying the Amazon as a region of particularly strong physical climate responsiveness, our analysis reveals the potential for particularly acute impacts arising from existing ecosystem vulnerability to the combined effects of severe heat and severe drought.

Impacts and vulnerability assessments have increasingly recognized the importance of multiple stresses in shaping vulnerability to climate change (Fussel and Klein 2006; Smith et al. 2009; Ford et al. 2010). Although our hotspot identification does not consider any of the non-climatic factors that will ultimately determine the impacts of climate change, by identifying regions that exhibit the greatest total change across a range of climate variables, our analysis can provide a compact, quantitative guide for identifying regions where multiple climate stresses could become pronounced, thereby motivating more detailed vulnerability assessment. While specific impacts will clearly be shaped by the interaction of climate change with human and biological vulnerabilities, our identification of climate change hotspots can help to inform mitigation and adaptation decisions by quantifying the rate, magnitude and causes of the aggregate climate response in different parts of the world.

## Electronic supplementary material

Below is the link to the electronic supplementary material.

ESM 1 (PDF 138 kb)

Figure S1 The change in each variable between the 1986-2005 and 2080-2099 periods. (JPEG 8270 kb)

Figure S1 High resolution image (EPS 77197 kb)

Figure S2 The relative magnitude of change in each variable between the 1986-2005 and 2080-2099 periods. The absolute values of change at each grid point are normalized to the maximum absolute value of all land grid points north of 60°S. (JPEG 6289 kb)

Figure S2 High resolution image (EPS 76063 kb)

Figure S3 The occurrence of the 1986-2005 maximum December-January-February (DJF) seasonal temperature in the 2016-2035, 2046-2065 and 2080-2099 periods of RCP8.5 (left) and RCP4.5 (right). The panels show the absolute occurrences as the percent of years in each 20-year period. The frequency of occurrence of the 1986-2005 maximum JJA seasonal temperature value is, by definition, 5% at each grid point during the 20-year 1986-2005 period. (JPEG 4280 kb)

Figure S3 High resolution image (EPS 19471 kb)

Table S1 Available models in the CMIP5 RCP8.5 ensemble archiving monthly surface air temperature (tas) and precipitation (pr) data for both the historical and 21st century periods. * (PDF 51.4 kb)
