# Supplementary material for: Climate change hotspots in the CMIP5 global climate model ensemble
Source: Clim Change. 2012 Aug 25;114(3):813–22. doi: 10.1007/s10584-012-0570-x (PMC3765072; doi:10.1007/s10584-012-0570-x)
Supplement: Supplementary file 1 — (PDF 138 kb) [file 10584_2012_570_MOESM1_ESM.pdf]

**Supplemental Methods**

*S1. Models*

We analyze global climate model output archived in Phase 5 of the Coupled Model Intercomparison Project (CMIP5) (Taylor et al. 2012). The CMIP5 ensemble is similar to the CMIP3 ensemble analyzed by Giorgi (2006), with a number of notable advances. First, whereas the CMIP3 ensemble was forced by atmospheric constituent concentrations from the SRES scenarios (IPCC 2000), the CMIP5 ensemble is forced by Representative Concentration Pathways (RCPs) (Moss et al. 2010). Second, whereas the CMIP3 ensemble was limited to atmosphere-ocean general circulation models (AOGCMs), the CMIP5 ensemble includes AOGCMs and "Earth system models" (ESMs) in which AOGCMs have been expanded to include interactive vegetation, biogeochemical cycles, and/or atmospheric chemistry. Third, a number of the models in the CMIP5 ensemble have been run at higher spatial resolution than the models in the CMIP3 ensemble.

Here we quantify climate change hotspots in 3 periods of the 21$^{st}$ century of RCP8.5 and RCP4.5 (2016-2035, 2046-2065, and 2080-2099). The suite of available RCP8.5 and RCP4.5 simulations includes realizations from 20 models, including 86 realizations of the baseline period (1986-2005), 51 realizations of the 21$^{st}$ century in the RCP8.5 pathway, and 51 realizations of the 21$^{st}$ century in the RCP4.5 pathway (Table S1).

*S2. Hotspot Quantification*

We extend the statistical approach of Diffenbaugh et al. (2008) and Williams et al. (2007) to quantify global climate change hotspots in the CMIP5 ensemble. Diffenbaugh et al. (2008) calculated the hotspot pattern in the United States using both the Standard Euclidean Distance (SED) and the Squared Chord Distance (SCD), and found the pattern to be robust to quantification method.

Here we focus on the SED, which quantifies the distance between two points in multi-dimensional space (Williams et al. 2007). We use the SED to calculate the total change in multi-dimensional climate space at each grid point between the present and future periods:

$$SED_{total} = (\sum_v SED_v)^{1/2} \quad \text{(Eq.1)}$$

for

$$SED_v = (abs(\Delta_v) / max[abs(\Delta_v)]_{ij})^2 \quad \text{(Eq.2)}$$

where $abs(\Delta_v)$ is the absolute value of change in climate indicator $v$ at each grid point between the present and future periods, and $max[abs(\Delta_v)]_{ij}$ is the maximum land-grid-point absolute value change in climate indicator $v$ over all land grid points $ij$ in the 2080-2099 period of RCP8.5.

### *S3. Climate Indicators*

We include 7 climate indicators in our quantification: mean surface air temperature (T), mean precipitation (P), interannual standard deviation of surface air temperature (Tvar), interannual coefficient of variation of mean precipitation (Pvar), occurrence of years above the baseline maximum surface air temperature (Hot), occurrence of years below the baseline minimum precipitation (Wet), and occurrence of

years above the baseline maximum precipitation (Dry). We treat each climate indicator separately in each of four seasons (DJF, MAM, JJA, SON), yielding 28 total climate dimensions for the SED calculation.

We calculate these 28 $\Delta_v$ changes between the baseline (1986-2005) and future periods (2016-2035, 2046-2065, and 2080-2099) at each grid point using the long-term ensemble mean of seasonal temperature ($T_{mean-e}$), seasonal precipitation ($P_{mean-e}$), interannual standard deviation of seasonal temperature ($T_{SD-e}$), interannual standard deviation of seasonal precipitation ($P_{SD-e}$), ensemble mean extreme hot years ($T_{hot-e}$), ensemble mean extreme wet years ($P_{wet-e}$), and ensemble mean extreme dry years ($P_{dry-e}$) for the DJF, MAM, JJA, and SON seasons at each 1-degree grid point for the baseline period (1986-2005) and each of the three future periods (2016-2035, 2046-2065, and 2080-2099):

Mean temperature: $\Delta_T = (T_{mean-e})_{future} - (T_{mean-e})_{baseline}$ (Eq.4)

Mean precipitation: $\Delta_P = ((P_{mean-e})_{future} - (P_{mean-e})_{baseline}) / (P_{mean-e})_{baseline}$ (Eq.5)

Temperature variability: $\Delta_{Tvar} = ((T_{SD-e})_{future} - (T_{SD-e})_{baseline}) / (T_{SD-e})_{baseline}$ (Eq.6)

Precipitation variability: $\Delta_{Pvar} = (((P_{SD-e})_{future} / (P_{mean-e})_{future}) - ((P_{SD-e})_{baseline} / (P_{mean-e})_{baseline})) / ((P_{SD-e})_{baseline} / (P_{mean-e})_{baseline})$ (Eq.7)

Extreme hot occurrence: $\Delta_{Hot} = (T_{hot-e})_{future}$ (Eq.8)

Extreme wet occurrence: $\Delta_{Wet} = (P_{wet-e})_{future}$ (Eq.9)

Extreme dry occurrence: $\Delta_{Dry} = (P_{dry-e})_{future}$ (Eq.10)

*S4. Normalization of Climate Indicators*

Because our goal is to identify climate change hotspots over the inhabited land areas, we mask out all grid points south of 60˚S and all ocean grid points north of 60˚S from each of the 28 climate indicators. We create a land mask from the soil moisture field of one model (CSIRO-Mk3-6-0) gridded to the common 1-degree grid (see description of regridding in section S5 below). We note that while this land mask is representative of the common 1-degree grid, it does not represent the exact land mask of each model gridded to the 1-degree grid.

Further, in order to treat the change in each of the 28 climate indicators equally in the SED calculation, we normalize the change in each climate indicator $v$ to the maximum land-grid-point change in that climate indicator that is found in the 2080-2099 period of RCP8.5.

To execute this normalization, we first calculate the $\Delta_v$ for each climate indicator in each of the three future periods, as described above in section S3.

We then calculate the absolute value of the $\Delta_v$ for each climate indicator $v$ in each of the three future periods:

$$(\Delta_v)_{abs} = \text{abs}(\Delta_v) \qquad \text{(Eq.11)}$$

We then calculate the maximum land-grid-point change $\max[\text{abs}(\Delta_v)]_{ij}$ found in the 2080-2099 period of RCP8.5 for each climate indicator $v$ over all land grid points $ij$ north of 60˚S:

$$(\Delta_v)_{max} = (\max[\text{abs}(\Delta_v)]_{ij})_{2080\text{-}2099} \qquad \text{(Eq.12)}$$

We then calculate the SED value for each climate indicator at each grid point as the square of the ratio between the absolute value of change in each climate indicator *v* and the maximum land-grid-point absolute value change in that climate indicator in the 2080-2099 period of RCP8.5:

$$SED_v = ((\Delta_v)_{abs} / (\Delta_v)_{max})^2 \qquad \text{(Eq.13)}$$

We then calculate the total SED as the square root of the sum of the 28 $SED_v$ values:

$$SED_{total} = (\text{sum}(SED_v))^{1/2} \qquad \text{(Eq.14)}$$

*S5. Calculation of the Ensemble Mean Climate Indicators*

As described above, we calculate the aggregate climate change from the ensemble mean of seasonal temperature ($T_{\text{mean-e}}$), interannual standard deviation of seasonal temperature ($T_{\text{SD-e}}$), seasonal precipitation ($P_{\text{mean-e}}$), and interannual standard deviation of seasonal precipitation ($P_{\text{SD-e}}$). We take the following steps to calculate these ensemble means from the individual realizations of the 20 models (Table S1):

1. First, following Giorgi (2006), Diffenbaugh et al. (2007), and Diffenbaugh et al. (2008), we interpolate the output from each realization *r* of each model *m* to a common 1-degree geographical grid. For models with horizontal resolution that is more coarse than 1 degree, we interpolate to the common 1-degree grid using bilinear interpolation. For models with horizontal resolution that is more fine than 1 degree, we interpolate to the common 1-degree grid using local area averaging (in order to account for fractional contributions from the high-resolution grid to the low-resolution grid).

2. Second, for each realization *r* of each model *m*, we calculate the yearly seasonal temperature ($T_{y\text{-}r}$) and precipitation ($P_{y\text{-}r}$) at each 1-degree grid point for the DJF, MAM, JJA, and SON seasons of each year *y* of the baseline period (1986-2005) and each year *y* of the three future periods (2016-2035, 2046-2065, and 2080-2099). (In order to maintain coherence of DJF seasons, we begin each year with the December of the preceding year.)

3. Third, for each realization *r* of each model *m*, we calculate the mean and standard deviation of the $T_{y\text{-}r}$ and $P_{y\text{-}r}$ values across the *y* years of that realization, yielding the long-term realization mean temperature ($T_{mean\text{-}r}$), realization mean precipitation ($P_{mean\text{-}r}$), realization interannual standard deviation of temperature ($T_{SD\text{-}r}$), and realization interannual standard deviation of precipitation ($P_{SD\text{-}r}$) for the DJF, MAM, JJA, and SON seasons at each 1-degree grid point in the baseline period (1986-2005) and each of the three future periods (2016-2035, 2046-2065, and 2080-2099):

$$T_{mean\text{-}r} = \text{mean}(T_{y\text{-}r}) \quad \text{(Eq.15)}$$

$$P_{mean\text{-}r} = \text{mean}(P_{y\text{-}r}) \quad \text{(Eq.16)}$$

$$T_{SD\text{-}r} = \text{stddev}(T_{y\text{-}r}) \quad \text{(Eq.17)}$$

$$P_{SD\text{-}r} = \text{stddev}(P_{y\text{-}r}) \quad \text{(Eq.18)}$$

Prior to calculating the interannual standard deviation of DJF, MAM, JJA, and SON seasonal temperature and precipitation, we remove the least squares linear trend of the 20-year seasonal timeseries ($T_{y\text{-}r}$ and $P_{y\text{-}r}$) at each 1-degree grid point.

4. Fourth, for each model *m*, we calculate the mean of the $T_{mean\text{-}r}$, $P_{mean\text{-}r}$, $T_{SD\text{-}r}$ and $P_{SD\text{-}r}$ values across all of the *r* realizations of that model, yielding the long-term model

mean temperature ($T_{mean\text{-}m}$), model mean precipitation ($P_{mean\text{-}m}$), model interannual standard deviation of temperature ($T_{SD\text{-}m}$), and model interannual standard deviation of precipitation ($P_{SD\text{-}m}$) for the DJF, MAM, JJA, and SON seasons at each 1-degree grid point for the baseline period (1986-2005) and each of the three future periods (2016-2035, 2046-2065, and 2080-2099):

$$T_{mean\text{-}m} = mean(T_{mean\text{-}r}) \quad \text{(Eq.19)}$$

$$P_{mean\text{-}m} = mean(P_{mean\text{-}r}) \quad \text{(Eq.20)}$$

$$T_{SD\text{-}m} = mean(T_{mean\text{-}r}) \quad \text{(Eq.21)}$$

$$P_{SD\text{-}m} = mean(P_{mean\text{-}r}) \quad \text{(Eq.22)}$$

5. Fifth, we calculate the mean of the $T_{mean\text{-}m}$, $P_{mean\text{-}m}$, $T_{SD\text{-}m}$ and $P_{SD\text{-}m}$ values across all of the *m* models in the CMIP5 ensemble, yielding the long-term ensemble mean temperature ($T_{mean\text{-}e}$), ensemble mean precipitation ($P_{mean\text{-}e}$), ensemble interannual standard deviation of temperature ($T_{SD\text{-}e}$), and ensemble interannual standard deviation of precipitation ($P_{SD\text{-}e}$) for the DJF, MAM, JJA, and SON seasons at each 1-degree grid point for the baseline period (1986-2005) and each of the three future periods (2016-2035, 2046-2065, and 2080-2099):

$$T_{mean\text{-}e} = mean(T_{mean\text{-}m}) \quad \text{(Eq.23)}$$

$$P_{mean\text{-}e} = mean(P_{mean\text{-}m}) \quad \text{(Eq.24)}$$

$$T_{SD\text{-}e} = mean(T_{mean\text{-}m}) \quad \text{(Eq.25)}$$

$$P_{SD\text{-}e} = mean(P_{mean\text{-}m}) \quad \text{(Eq.26)}$$

6. Sixth, for each realization *r* of each model *m*, we calculate the baseline maximum seasonal temperature ($T_{max-r}$), maximum seasonal precipitation ($P_{max-r}$), and minimum seasonal precipitation ($P_{min-r}$) at each 1-degree grid point for the DJF, MAM, JJA, and SON seasons from the baseline (1986-2005) $T_{y-r}$ and $P_{y-r}$ seasonal timeseries described in (2):

$$T_{max-r} = \max(T_{y-r}) \quad \text{(Eq.27)}$$

$$P_{max-r} = \max(P_{y-r}) \quad \text{(Eq.28)}$$

$$P_{min-r} = \min(P_{y-r}) \quad \text{(Eq.29)}$$

7. Seventh, for each model *m*, we calculate the mean of the $T_{max-r}$, $P_{max-r}$, and $P_{min-r}$ values across all of the *r* realizations of that model, yielding the long-term model mean maximum temperature ($T_{max-m}$), model mean maximum precipitation ($P_{max-m}$), and model mean minimum precipitation ($P_{min-m}$) for the DJF, MAM, JJA, and SON seasons at each 1-degree grid point for the baseline period (1986-2005):

$$T_{max-m} = \text{mean}(T_{max-r}) \quad \text{(Eq.30)}$$

$$P_{max-m} = \text{mean}(P_{max-r}) \quad \text{(Eq.31)}$$

$$P_{min-m} = \text{mean}(P_{min-r}) \quad \text{(Eq.32)}$$

8. Eighth, for each realization *r* of each model *m*, we calculate: (a) the number of years in which the DJF, MAM, JJA, and SON seasonal temperature $T_{y-r}$ is greater than the respective model mean baseline $T_{max-r}$ value at each 1-degree grid point in each of the three future periods (2016-2035, 2046-2065, and 2080-2099), yielding the long-term realization mean extreme hot seasons ($T_{hot-r}$); (b) the number of years in which the DJF,

MAM, JJA, and SON seasonal precipitation $P_{y\text{-}r}$ is greater than the respective model mean baseline $P_{max\text{-}m}$ value at each 1-degree grid point in each of the three future periods (2016-2035, 2046-2065, and 2080-2099), yielding the long-term realization mean extreme wet years ($P_{wet\text{-}r}$); and (c) the number of years in which the DJF, MAM, JJA, and SON seasonal precipitation Py-r is less than the respective model mean baseline $P_{min\text{-}m}$ value at each 1-degree grid point in each of the three future periods (2016-2035, 2046-2065, and 2080-2099), yielding the long-term realization mean extreme dry years ($P_{dry\text{-}r}$):

$$T_{hot\text{-}r} = \mathrm{sum}(T_{y\text{-}r} > T_{max\text{-}m}) \quad \text{(Eq.33)}$$

$$P_{wet\text{-}r} = \mathrm{sum}(P_{y\text{-}r} > P_{max\text{-}m}) \quad \text{(Eq.34)}$$

$$P_{dry\text{-}r} = \mathrm{sum}(P_{y\text{-}r} < P_{min\text{-}m}) \quad \text{(Eq.35)}$$

9. Ninth, for each model *m*, we calculate the mean of the $T_{hot\text{-}r}$, $P_{wet\text{-}r}$, and $P_{dry\text{-}r}$ values across all of the *r* realizations of that model, yielding the long-term model mean extreme hot years ($T_{hot\text{-}m}$), model mean extreme wet years ($P_{wet\text{-}m}$), and model mean extreme dry years ($P_{dry\text{-}m}$) for the DJF, MAM, JJA, and SON seasons at each 1-degree grid point for each of the three future periods (2016-2035, 2046-2065, and 2080-2099):

$$T_{hot\text{-}m} = \mathrm{mean}(T_{hot\text{-}r}) \quad \text{(Eq.36)}$$

$$P_{wet\text{-}m} = \mathrm{mean}(P_{wet\text{-}r}) \quad \text{(Eq.37)}$$

$$P_{dry\text{-}m} = \mathrm{mean}(P_{dry\text{-}r}) \quad \text{(Eq.38)}$$

10. Tenth, we calculate the mean of the $T_{hot\text{-}m}$, $P_{wet\text{-}m}$, and $P_{dry\text{-}m}$ values across all of the *m* models of the CMIP5 ensemble, yielding the long-term ensemble mean extreme hot years ($T_{hot\text{-}e}$), ensemble mean extreme wet years ($P_{wet\text{-}e}$), and ensemble mean extreme

dry years ($P_{dry\text{-}e}$) for the DJF, MAM, JJA, and SON seasons at each 1-degree grid point for each of the three future periods (2016-2035, 2046-2065, and 2080-2099):

$$T_{hot\text{-}e} = \text{mean}(T_{hot\text{-}m}) \quad \text{(Eq.39)}$$

$$P_{wet\text{-}e} = \text{mean}(P_{wet\text{-}m}) \quad \text{(Eq.40)}$$

$$P_{dry\text{-}e} = \text{mean}(P_{dry\text{-}m}) \quad \text{(Eq.41)}$$

These ten steps yield the ensemble mean of the 28 climate indicators from which we calculate the aggregate climate change between the baseline (1986-2005) and future periods (2016-2035, 2046-2065, and 2080-2099).

**Supplemental References**


Diffenbaugh, N. S., F. Giorgi, et al. (2008). Climate change hotspots in the United States. *Geophysical Research Letters* **35**(in press): doi:10.1029/2008GL035075.

Diffenbaugh, N. S., F. Giorgi, et al. (2007). Indicators of 21st century socioclimatic exposure. *Proceedings of the National Academy of Sciences of the United States of America* **104**(51): 20195-20198.

Giorgi, F. (2006). Climate change hot-spots. *Geophysical Research Letters* **33**(8): L08707, doi:08710.01029/02006GL025734.

Giorgi, F. (2008). A simple equation for regional climate change and associated uncertainties. *Journal of Climate* **21**: 1589-1604.

Giorgi, F. and X. Bi (2005). Updated regional precipitation and temperature changes for the 21st century from ensembles of recent AOGCM simulations. *Geophysical Research Letters* **32**: L21715, doi:21710.21029/22005GL024288.

IPCC, W. G. I. (2000). *Special Report on Emissions Scenarios*. Cambridge, UK, Cambridge University Press.

Moss, R. H., J. A. Edmonds, et al. (2010). The next generation of scenarios for climate change research and assessment. *Nature* **463**(7282): 747-756.

Rogelj, J., M. Meinshausen, et al. (2012). Global warming under old and new scenarios using IPCC climate sensitivity range estimates. *Nature Clim. Change* **advance online publication**.

Taylor, K. E., R. J. Stouffer, et al. (2012). An overview of CMIP5 and the experiment design. *Bulletin of the American Meteorological Society* **93**(4): 485-498.

Williams, J. W., S. T. Jackson, et al. (2007). Projected distributions of novel and disappearing climates by 2100AD. *Proceedings of the National Academy of Sciences* **104**(14): 5738-5742.
