# Supplementary figures and images for: Climate change hotspots in the CMIP5 global climate model ensemble
Source: Clim Change. 2012 Aug 25;114(3):813–22. doi: 10.1007/s10584-012-0570-x (PMC3765072; doi:10.1007/s10584-012-0570-x)

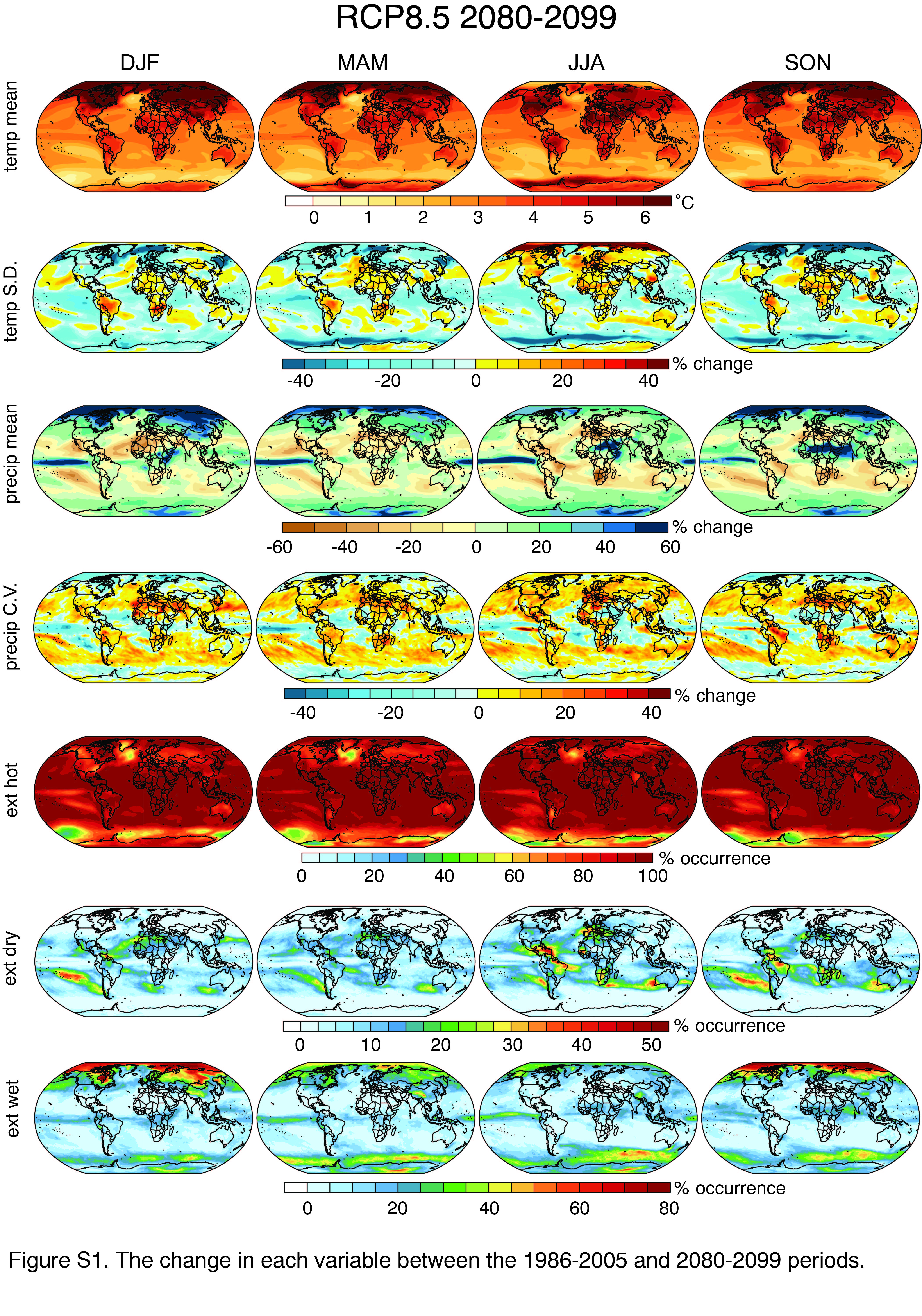

Supplement: Supplementary file 2 — The change in each variable between the 1986-2005 and 2080-2099 periods. (JPEG 8270 kb) [file 10584_2012_570_Fig4_ESM.jpg]

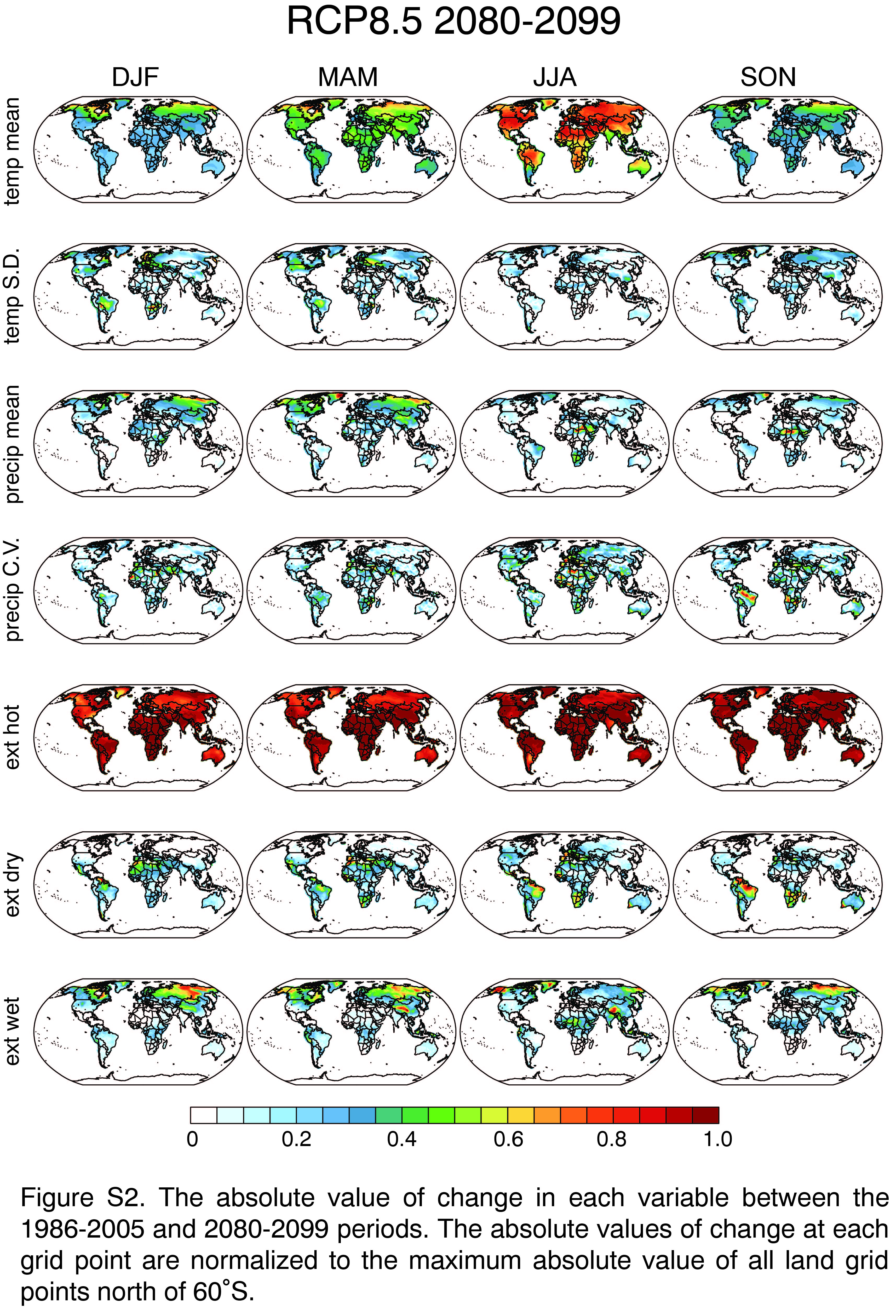

Supplement: Supplementary file 4 — The relative magnitude of change in each variable between the 1986-2005 and 2080-2099 periods. The absolute values of change at each grid point are normalized to the maximum absolute value of all land grid points north of 60°S. (JPEG 6289 kb) [file 10584_2012_570_Fig5_ESM.jpg]

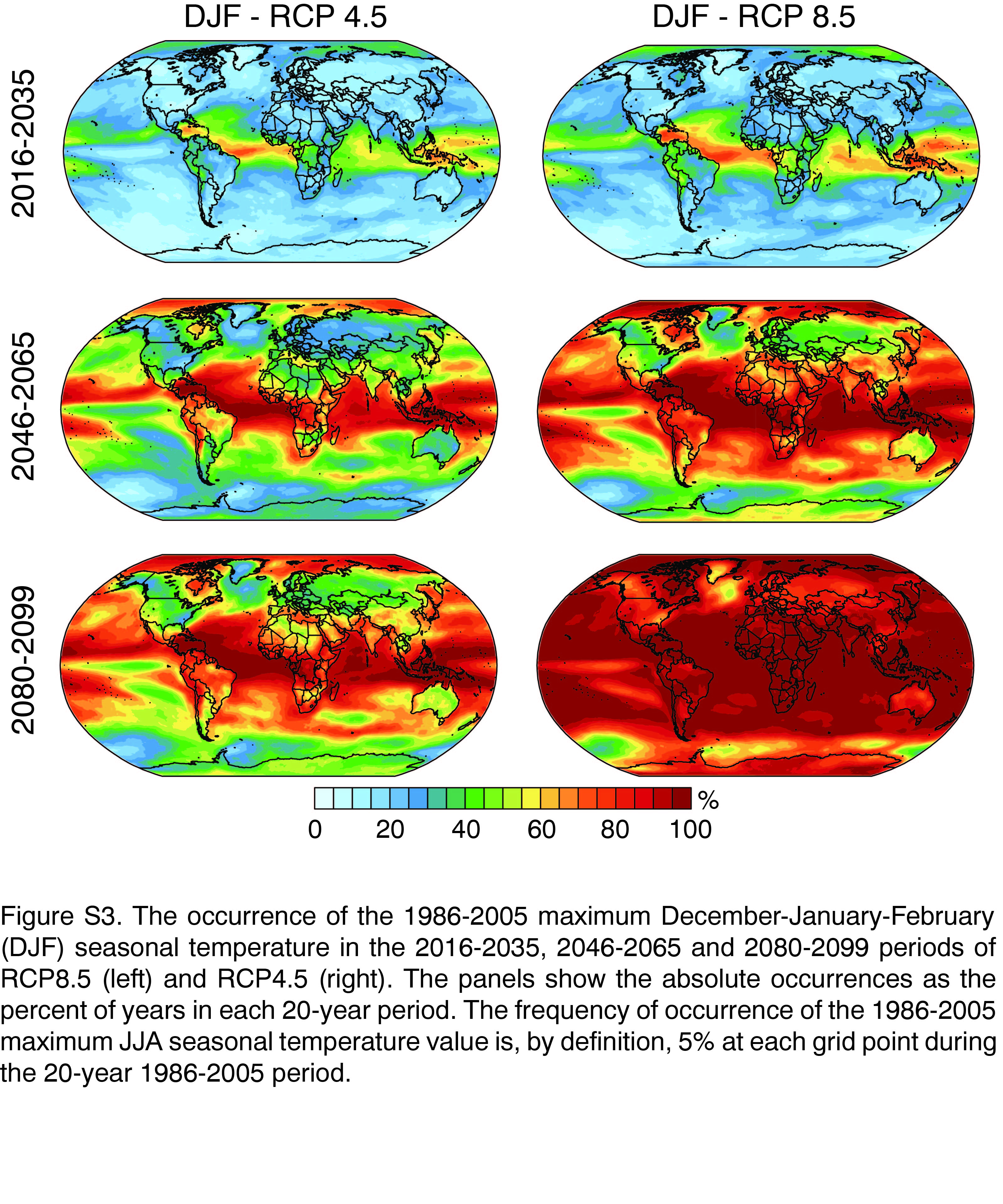

Supplement: Supplementary file 6 — The occurrence of the 1986-2005 maximum December-January-February (DJF) seasonal temperature in the 2016-2035, 2046-2065 and 2080-2099 periods of RCP8.5 (left) and RCP4.5 (right). The panels show the absolute occurrences as the percent of years in each 20-year period. The frequency of occurrence of the 1986-2005 maximum JJA seasonal temperature value is, by definition, 5% at each grid point during the 20-year 1986-2005 period. (JPEG 4280 kb) [file 10584_2012_570_Fig6_ESM.jpg]
