# Supplementary material for: Climate change hotspots in the CMIP5 global climate model ensemble
Source: Clim Change. 2012 Aug 25;114(3):813–22. doi: 10.1007/s10584-012-0570-x (PMC3765072; doi:10.1007/s10584-012-0570-x)
Supplement: Supplementary file 8 — Available models in the CMIP5 RCP8.5 ensemble archiving monthly surface air temperature (tas) and precipitation (pr) data for both the historical and 21st century periods. * (PDF 51.4 kb) [file 10584_2012_570_MOESM5_ESM.pdf]

Table S1. Available models in the CMIP5 RCP8.5 ensemble archiving monthly surface air temperature (tas) and precipitation (pr) data for both the historical and 21st century periods. *

**Model**	**Historical Realizations**	**RCP8.5 Realizations**	**RCP4.5 Realizations**
ACCESS1-0	1	1	1
bcc-csm1-1	3	1	1
CanESM2	5	5	5
CCSM4	6	6	5
CNRM-CM5	10	5	1
CSIRO-Mk3-6-0	10	10	10
FGOALS-g2	4	1	1
GFDL-ESM2G	1	1	1
GFDL-ESM2M	1	1	1
GISS-E2-R	16	1	5
HadGEM2-CC	3	3	1
HadGEM2-ES	4	1	4
inmcm4	1	1	1
IPSL-CM5A-LR	4	4	4
MIROC5	4	3	3
MIROC-ESM-CHEM	1	1	1
MIROC-ESM	1	1	1
MPI-ESM-LR	3	3	3
MRI-CGCM3	5	1	1
NorESM1-M	3	1	1

* see Supplemental Information for description of approach to averaging across the ensemble
